# Trends in burden of chronic obstructive pulmonary disease in Iran, 1995–2015: findings from the global burden of disease study

**DOI:** 10.1186/s13690-020-00426-x

**Published:** 2020-05-25

**Authors:** Seyed Yaser Hashemi, Victoria Momenabadi, Ahmad Faramarzi, Amin Kiani

**Affiliations:** 1 Fasa University of Medical Sciences, Fasa, Iran; 2 Department of Public Health, Bam University of Medical Sciences, Bam, Iran., Bam University of Medical Sciences, Bam, Iran; 3 Department of Health Management and Economics, School of Public Health, Urmia University of Medical Sciences, Urmia, Iran; 4 School of Public Health, Fasa University of Medical Sciences, Fasa, Iran

**Keywords:** COPD, DALY, Prevalence, Incidence, Mortality

## Abstract

**Background:**

Chronic obstructive pulmonary disease (COPD) is a heterogeneous disorder that progresses over time, and currently it is the fourth leading cause of death across the globe. The World Health Organization (WHO) predicts that the disease will become the third leading cause of death by 2030. **The present study aimed to assess the burden trends of COPD in Iran by estimating the disability-adjusted life years (DALYs) from 1995 to 2015.**

**Methods:**

Data were retrospectively collected as the Global Burden of Disease (GBD) from 1995 to 2015 and published by the Institute for Health Metrics and Evaluation. We applied DALYs, incidence and prevalence rate to report the burden of COPD in Iran. To assess the statistical significance according to trend, the Cochran-Armitage test was applied. **Additionally, the t-test was used to analyze the DALYs number by gender and Onaway ANOVA by age groups at a significance level set at*P*** **< 0.05.**

**Results:**

From 1995 to 2015, there were approximately **1.1 million DALYs** attributable to COPD in Iran. In both genders and at all ages, the number of DALYs increased significantly from **176,224** in 1995 to **253,618** in 2015. The incidence and prevalence rate were 76.65 and 1491.37 per 100,000 population, respectively in both genders in 2015 in Iran. It is noticeable that the number of deaths during the study years, 1995 to 2015, was **39,064. This study showed that the COPD burden was significantly different by age groups and gender.**

**Conclusions:**

**COPD is still a public health problem in Iran and has an increasing trend**. The majority of DALYs were due to **the years of life lost as a result of premature death (YLLs),** indicating that prevention and early detection, especially in the age groups of 15 to 70 years, should be considered.

## Background

Chronic obstructive pulmonary disease (COPD) is a heterogeneous disorder that progresses over time, and characterized by airflow limitation that is not fully reversible [1, 2]. Recently, COPD has grown dramatically, making it the fourth leading cause of death across the globe. In this regard, the World Health Organization (WHO) predicts that the disease will become the third leading cause of death by 2030 [3]. The prevalence of COPD is increasing due to urbanization, industrial pollution, tanneries and biomass fuel burning inside homes, especially in Asian and African countries [4]. A study calculated a mean prevalence of 13.4% for COPD in Africa, ranging from 9.4 to 22.1% [5]. However, in Asia, its prevalence was reported 13.5% with a range of 3 to 22.2% [6].

Furthermore, COPD is a common reason for hospital admission in many countries, playing a crucial role in imposing health care costs [7]. A systematic review demonstrated that the annual hospitalization cost per patient for the COPD in the United States was $6852, and in Iran and China, it was estimated as $865 and $1477, respectively [8]. Another study reported a health care expenditure of £781 to £1639 in the United Kingdom, per patient per year [9].

The Global Burden of Disease (GBD) study provides estimates of a burden of disease, an approach summarizing the effects of morbidity and premature mortality in a special population, in different countries and regions [10, 11]. To estimate disease trends, the burden of diseases is employed in different indicators such as incidence, prevalence, duration and mortality [12, 13]. Among the specified indexes, we emphasized on disability-adjusted life years (DALYs) to measure the burden of COPD in Iran.

Although, COPD has imposed an extensive burden on the health, life expectancy, and well-being of the population in low and middle-income countries like Iran. Based on the GBD study, there were 40,141 incidence cases of COPD in 1995 in Iran, and the incidence rate was 60,587 in 2015 [14]. Most studies into the burden of COPD have been conducted in developed countries, and there is no study in Iran. Therefore, the main purpose of this study was to assess the burden trends of COPD in Iran by estimating the years of life lost due to premature death (YLLs) and the years lived with disability (YLDs) in patients with COPD. Moreover, indicators such as number of deaths, prevalence, incidence, and mortality rate were evaluated.

## Materials and methods

### Data collection

The data of the number of DALYs for COPD were derived from the results of the GBD study. The data were extracted during 1995 to 2015. The GBD study was published by the Institute for Health Metrics and Evaluation (IHME). The first report of the GBD study was published in 1990, and it has been published annually to the present. The GBD study has recruited a consortium of more than 3600 researchers in more than 145 countries to collect and analyze data. The initial data on the GBD study are premature death and disability from more than 350 diseases and injuries in 195 countries by age and sex. The GBD study applies different sources to assess the outcomes of health loss. These sources are the death registration systems, vital registration, verbal autopsy, mortality surveillance and others. A complete description of the data, statistical modeling, and metrics has been specified in the references [15, 16].

### Data analysis

The current research is a secondary analysis of the GBD study results. To evaluate the trend of COPD burden in Iran, we used mortality and morbidity indices. The number of deaths, incidence and prevalence rate, YLLs, YLDs, and DALYs are reported for both sexes and all age groups from 1995 to 2015. In the GBD study, the results were reported based on different age grouping; we applied the method proposed by the WHO and other studies. These groups include under 5 years, 5–14 years, 15–49 years, 50–69 years, and over 70 years.

In this study, we highlighted DALYs and their component to measure the burden of COPD in Iran DALY is a tool developed by the World Health Organization (WHO) to measure, compare, and analyze the burden of various diseases [11]. This metric combines the years of life lost due to premature death (YLLs) and the time lived in states of less than optimal health, loosely referred to as disability (YLDs), for a special cause in a given year. A DALY is equal to the loss of 1 year of “healthy” life from the combined impacts of mortality and disability. In fact, DALY for the particular sex and age is taken by summing YLLs and YLDs in a given year [17, 18]. In the GBD study, YLLs are considered a measure of cause-specific premature mortality. Therefore, for a given cause, age, and sex, this metric is equal to the death number multiplied by the standard life expectancy [19]. YLDs were derived from the multiplication of the incidence number for a specific reason by the duration of disability and a weight factor [20].

To more accurately explain the burden of COPD in Iran, we performed the following steps.

First, given to different classifications on COPD, we used the 10th version of the International Classification of Diseases (ICD). These diseases fall into the category of chronic respiratory diseases, and it includes emphysema means an anatomical condition characterized by destruction and enlargement of the lung alveoli, chronic bronchitis, which is a clinical condition with chronic cough and phlegm, and small airways disease, a condition in which small bronchioles are narrowed. Then, number of deaths, prevalence and incidence rate, DALYs number, YLLs and YLDs were reported to obtain a description of COPD. Second, the trends in the burden of COPD were investigated. We assessed the trends by gender and age groups. To assess statistical significance according to trend, the Cochran-Armitage test was applied. Additionally, the t-test was used for analysis, since the mean of variables was compared between the two groups of males and females. Furthermore, one-way ANOVA analysis was applied to assess the burden according to age groups. The significance level was set at 0.05. We used the Stata version 13 and Microsoft Office Excel 2013 programs to perform our analysis.

## Results

In the following, we report noticeable findings from the GBD attributed to the burden of COPD in Iran from 1995 to 2015. The number of DALYs, YLLs, YLDs are described. Moreover, death number, incidence, and prevalence rate are reported.

### Death number, incidence and prevalence rate

In Iran, there were 39,064 deaths in both genders from 1995 to 2015 due to COPD. The number of deaths was 4899 in 1995 and 10,514 in 2015 for both sexes. The incidence rate for both genders was increased from 66.32 per 100,000 population in 1995 to 76.65 in 2015. The prevalence rate increased from 1247.87 in 1995 to 1491.37 per 100,000 population in 2015 in both genders. Furthermore, the results of this study indicated that number of deaths, incidence, and prevalence rate were significantly higher in males than in females (*p*-value< 0.05). Table 1 shows the number of deaths, incidence and prevalence rate for COPD according to years and age groups.

**Table 1:** The number of deaths, incidence and prevalence rate of COPD in Iran from 1995 to 2015

Years	Death number				Incidence per 100,000				Prevalence per 100,000			
	Male	Female	Both	*P* value	Male	Female	Both	*P* value	Male	Female	Both	*P* value
1995	2977	1922	4899	0.009*	77.83	54.47	66.32	0.004*	1403.26	1087.96	1247.87	0.008*
2000	3481	2209	5690		79.89	52.24	66.25		1486.94	1090.93	1291.6	
2005	5058	3149	8208		83.25	51.3	67.61		1578.72	1102.31	1345.45	
2010	5937	3816	9753		90.18	53.97	72.28		1702.5	1144.46	1426.76	
2015	6981	3532	10,514		96.33	56.69	76.65		1785.17	1193.12	1491.37	

*The *P*-value indicates statistical significance between male and female groups for death number, crude incidence, and prevalence rate

### DALYs, YLLs and YLDs

Table 2 presents the number of DALYs and deaths due to COPD in Iran from 1995 to 2015 based on age groups and sex. From 1995 to 2015, it was estimated that there were 1,059,491 DALYs of COPD for all ages in Iran, of which 652,409 DALYs occurred for males and 407,082 DALYs for females. The number of deaths was calculated 39,064 for all ages. The age group 50–69 years with 352,594 years had the highest DALYs, followed by the age group 15–49 years with 304,357 years.

**Table 2:** The death and DALYs number of COPD in different age groups in Iran from 1995 to 2015

	DALYs number			Death number		
Age groups	Female	Male	Total	Female	Male	Total
**Under 5**	41,161	43,947	85,108	468	502	970
**5–14 years**	13,260	13,149	26,409	61	72	133
**15–49 years**	115,857	188,500	304,357	1272	2580	3852
**50–69 years**	126,499	226,095	352,594	3896	7234	11,130
**> 70 years**	110,303	180,720	291,023	8932	14,047	22,979
**All ages**	407,082	652,409	1,059,491	14,630	24,434	39,064

The number of DALYs due to COPD in Iran for all ages was 176,224 in 1995 and, it was increased to 253,618 in 2015. >Throughout the years, the number of DALYs on COPD was associated with YLLs to YLDs. At all ages and in both genders, the YLLs were 137,461 in 1995 and 203,558 in 2015. Whereas, the YLDs were 38,761 in 1995 and 50,058 in 2015 (Table 3). The results also revealed that the burden of COPD in Iran had a significant trend from 1995 to 2015.

**Table 3:** YLLs and YLDs of COPD in different age groups in Iran from 1995 to 2015

		YLLs		YLDs		DALYs			Cochran-Armitage test	Onaway ANOVA
age	year	Female	Male	Female	Male	Female	Male	Both		
Under 5	1995	17,148	16,553	325	265	17,474	16,819	34,293	Z = 2 *P* value = 0.04*	F = 19 *P* value = 0.005*
5–14 years	1995	1602	1784	2771	2317	4374	4101	8476		
15–49 years	1995	11,464	19,259	9342	10,202	20,807	29,462	50,270		
50–69 years	1995	15,367	26,653	3853	5422	19,221	32,076	51,298		
> 70 years	1995	10,705	16,921	1796	2462	12,502	19,383	31,885		
All ages	1995	56,289	81,172	18,090	20,671	74,380	101,843	176,224		
Under 5	2000	11,563	13,516	213	183	11,776	13,700	25,476		
5–14 years	2000	1245	1474	2222	1994	3467	3469	6936		
15–49 years	2000	12,677	23,403	10,042	11,741	22,719	35,145	57,864		
50–69 years	2000	16,549	28,074	3911	5637	20,460	33,712	54,173		
> 70 years	2000	13,851	21,512	2132	2977	15,984	24,489	40,473		
All ages	2000	55,886	87,982	18,521	22,533	74,407	110,516	184,924		
Under 5	2005	6221	7634	151	138	6373	7772	14,145		
5–14 years	2005	869	1103	1427	1318	2297	2421	4719		
15–49 years	2005	12,850	25,606	10,358	12,820	23,209	38,427	61,637		
50–69 years	2005	19,916	34,580	4143	6115	24,059	40,696	64,755		
> 70 years	2005	23,377	37,068	2528	3798	25,905	40,867	66,773		
All ages	2005	63,235	105,994	18,609	24,191	81,844	130,186	212,031		
Under 5	2010	2972	2950	136	130	3109	3081	6191		
5–14 years	2010	552	645	1047	1020	1599	1666	3265		
15–49 years	2010	13,832	28,581	10,796	13,648	24,629	42,229	66,858		
50–69 years	2010	23,762	43,469	5003	7007	28,765	50,476	79,242		
> 70 years	2010	27,823	41,995	2974	4340	30,798	46,336	77,134		
All ages	2010	68,943	117,642	19,959	26,148	88,902	143,790	232,693		
Under 5	2015	2291	2442	137	130	2428	2573	5002		
5–14 years	2015	441	505	1080	938	1521	1489	3011		
15–49 years	2015	13,297	29,139	11,194	14,094	24,492	43,234	67,726		
50–69 years	2015	27,742	60,296	6248	8835	33,991	69,132	103,123		
> 70 years	2015	22,162	45,239	2950	4402	25,113	49,642	74,755		
All ages	2015	65,934	137,624	21,611	28,447	87,546	166,071	253,618		

The Cochran-Armitage test indicates that the DALYs number of COPD has a significant trend from 1995 to 2015. The Onaway ANOVA shows a significant difference in the number of DALYs between age groups

**Fig. 1:**
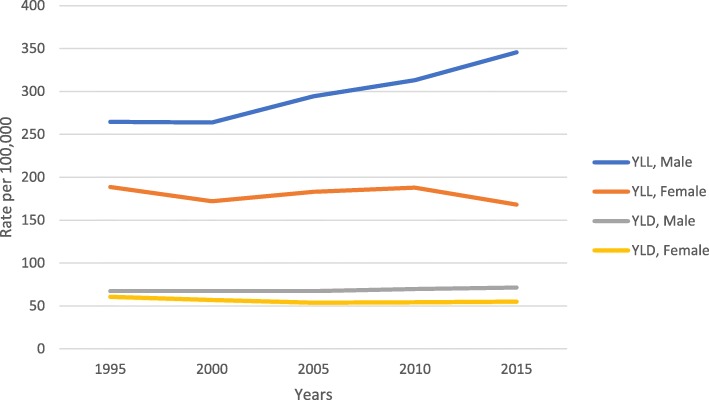
The rate of DALY per 100,000 of COPD by sex in Iran from 1995 to 2015

Our findings demonstrated the burden of COPD was higher in males than in females. The rate of YLLs was 265 and 189 per 100,000 population for males and females, respectively. Nevertheless, the rate was increased to 345 per 100,000 population in males in 2015 and was decreased to 168 in females. Figure 1 shows the rate of YLLs and YLDs per 100,000 population on COPD by gender.

**Fig. 2:**
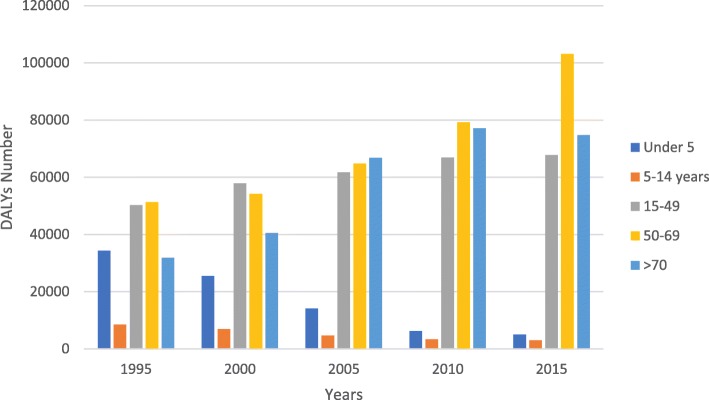
The DALY numbers of COPD in different age groups by years in Iran from 1995 to 2015

This study indicates that the maximum of the DALYs number between 1995 and 2015 was associated with the age groups of 50–69, 15–49 and > 70 years. Figure 2 illustrates the DALYs number across different age groups. In 2015, the DALYs number on the age group 50–69 years for both genders was 103.1 thousand, 74.8 thousand for the group > 70, and 67.7 thousand in the age group 15–49 years. As Fig. 2 shows, the group under 5-year has a diminishing trend in the DALYs number from 1995 to 2015.

## Discussion

There were various indicators applied to study the burden of diseases such as number of deaths, prevalence and incidence rate, DALY, YLL, and YLD. In this study, we investigated the burden of COPD according to the results of the GBD study in Iran and set an applicable pattern for COPD burden. Approximately 10,514 deaths and 253.6 thousand DALYs of COPD were estimated, and the incidence and prevalence rate were 76.6 and 1491 per 100,000 population, respectively, in both genders in Iran in 2015. Interestingly, the number of deaths during the study years, 1995 to 2015, was 39.1 thousand, and the number of DALYs was approximately 1.1 million. These estimates indicate that the number of DALYs due to COPD in Iran accounts for 1.2% of total DALYs in 2015, and the number of COPD deaths in Iran includes almost 2.8% of all deaths. Moreover, this study demonstrates that the COPD burden in Iran is more than that of some countries. For example, the burden of COPD in African countries was responsible for 0.3% of all DALYs, and 1.1% of all DALYs in high-mortality countries in American countries. However, there are many countries with a higher burden of COPD compared to Iran. The number of DALYs due to COPD in South-East Asia consists of 2.3 and 1.9% of all DALYs in countries with low and high mortality, respectively. Furthermore, COPD causes approximately 2% of the entire global burden of diseases [21].

According to the GBD results, the age group of 50–69 years incurred the highest COPD burden in Iran. From 1995 to 2015, this group approximately accounted for 33% of the number of COPD DALYs. Additionally, 28.5% of the deaths from COPD occurred in this group. The age groups 15–49 years and > 70 years were responsible for 28 and 27% of the number of DALYs, respectively. There are several reasons that the COPD burden mostly occurs between the ages of 15 to 70 years. First, studies have shown that the risk of death for COPD is higher in this population. For example, a study illustrated that the relative risk of mortality for the age group of 45–49 years was more than that for other groups [21]. One study by Gashaw et al. in Ethiopia showed that more than 60% of cases for COPD occurred in individuals over 30 years [22]. Secondly, part of the higher burden of COPD in this age group is due to the population structure of Iran, so that the population of 20 to 60 years in Iran has increased in recent years. Third, it may be owing to an increase in exposure to factors such as air pollution, cigarette, smoking, and occupational conditions that are more common in these groups. May et al. examined the burden of COPD in 2015. Their results explicated that in the United States, in the last 20 years, COPD accounted for 56.7% of deaths related to lung diseases, and deaths from COPD increased, especially in females. The mortality relative risk from COPD in smokers compared to nonsmokers is 25.61 for males and 22.35 for females with mortality continuing to rise in both genders [23].

The present study clearly demonstrated that why the burden of COPD was higher in males than in females, such that the number of DALYs for males was 60% more than that for females at all ages from 1995 to 2015. Furthermore, the number of deaths for males was 67% more than that for females, which was statistically significant. Minicuci et al studied the DALYs due to COPD in northern Italy in 2011 and estimated that total DALY (per 1000) for COPD varied between 2.1 and 3.4 years among males and between 1 and 2.3 years among females [24]. A study in Finland indicated that 78% of the samples who had a symptom of COPD were males [25]. The higher burden of COPD in males can be due to several reasons. COPD occurs more frequently in males. In all years, the number of deaths among males was more than that among females. The number of deaths was 6981 and 3532 for males and females, respectively, in 2015. Moreover, the incidence and prevalence rate were higher in males than in females. Studies revealed more risk factors for COPD in males than in females [26, 27]. Part of the increased burden of COPD in males is related to population structure and demographic characteristics. In Iran, the population of males is higher than that of females, and the employment rate is higher for males.

As other studies, our results demonstrated that **YLLs** were the main contributor to DALYs calculations, since COPD was a **mortal** disease. In fact, the burden of fatal diseases had predominantly affected from premature death. The assessed DALY showed that this parameter had an increasing process from 1995 to 2015 at all ages and in both genders. In 2015 compared to 1995, there was an approximate increase of 43% in the DALYs number due to COPD. While the number of deaths at 114%, the YLLs by 48%, and the YLDs on 29% were increased. Moreover, these results disclose that the burden of COPD in Iran, like other countries, has raised significantly over time. Studies have estimated that COPD will become the third most common cause of death by the year 2020, leading to being an important cause of morbidity, mortality, and health-care costs worldwide [4, 28].

To the best of our knowledge, this is the first attempt assessing the burden of COPD in Iran. The morbidity and mortality indices concurrently reported that it could be a road map for health policymakers. In addition, analysis of the burden of COPD based on a trend can provide useful information to estimate the economic burden of this disease. However, this study had some limitations. The results of the GBD study do not distinguish between the results of diseases related to COPD and assess them in a category; this is highly important for policy purposes.

## Conclusions

DALY represents a summary measure to improve the capacity of assessing population health needs and priorities. Policymakers owning such a further element of evaluation may be better oriented in allocating resources for COPD among the different health care chapters: prevention, emergency, chronicity, and rehabilitation. COPD is a critical public health issue in Iran and worldwide. It has had an increasing trend in recent years. The results of this study and others indicated that the majority of DALYs in COPD were due to YLLs, demonstrating that prevention and early detection should be considered, especially in the age group of 15 to 70 years.

## Data Availability

The preliminary data on chronic obstructive pulmonary disease are available in the global burden of disease study.
